# COVID-19 and stroke—Understanding the relationship and adapting services. A global World Stroke Organisation perspective

**DOI:** 10.1177/17474930211005373

**Published:** 2021-04-13

**Authors:** Hugh S Markus, Sheila Martins

**Affiliations:** 1 Stroke Research Group, Department of Clinical Neurosciences, University of Cambridge, Cambridge, UK; 2 Department of Neurology, Hospital de Clínicas de Porto Alegre, Universidade Federal do Rio Grande do Sul and Hospital Moinhos de Vento, Porto Alegre, Brazil

**Keywords:** COVID-19, stroke, pandemic, World Stroke Organization, healthcare systems, Telemedicine

## Abstract

A year ago the World Stroke Organisation (WSO) highlighted the enormous global impact of the COVID-19 pandemic on stroke care. In this review, we consider a year later where we are now, what the future holds, and what the long-term effects of the pandemic will be on stroke. Stroke occurs in about 1.4% of patients hospitalized with COVID-19 infection, who show an excess of large vessel occlusion and increased mortality. Despite this association, stroke presentations fell dramatically during the pandemic, although emerging data suggest that total stroke mortality may have risen with increased stroke deaths at home and in care homes. Strategies and guidelines have been developed to adapt stroke services worldwide, and protect healthcare workers. Adaptations include increasing use of telemedicine for all aspects of stroke care. The pandemic is exacerbating already marked global inequalities in stroke incidence and mortality. Lastly, the pandemic has had a major impact on stroke research and funding, although it has also emphasized the importance of large scale collaborative research initiatives.

## Introduction

It is a year since the World Stroke Organisation (WSO) highlighted the enormous global impact of the COVID-19 pandemic on stroke care. Not only was there the worrying concern that COVID-19 infection itself may increase stroke risk, but a WSO survey highlighted the impact of the pandemic on stroke care with delayed presentation and reduced hospital admission, and less availability of specialized stroke services due to diversion of resources to the care of COVID-19 patients.^1^ The last year has seen intense efforts to better understand the relationship between COVID-19 and stroke, to adapt stroke services to ensure adequate stroke care despite the constraints of the pandemic, and to ensure safety of both health professionals and patients. It is timely to consider where we are now, what the future holds, and what the long-term effects on stroke will be.

## Does COVID-19 cause stroke?

A recent systematic review and meta-analysis of 108,571 COVID-19 patients, in 1106 of whom ischemic or hemorrhagic stroke occurred, yielded an overall pooled incidence of acute stroke in COVID-19 patients of 1.4% (95% CI 1.0–1.9).^2^ What proportion of these strokes are causally related to COVID infection, and what proportion are incidentally related, is still uncertain. However, increasing data suggest that at least in a proportion, the two are causally related. Evidence supporting a causal relation comes from an epidemiological study that found COVID-19 is associated with a 7.6-fold increased risk in the odds of stroke compared with influenza.^3^ Furthermore, a characteristic pattern of stroke is seen in many COVID-19 patients. Stroke patients with COVID-19 were younger and less likely to have hypertension and previous stroke, than stroke patients without COVID-19. Large artery occlusion is much more common in COVID-19 associated stroke, and a characteristic pattern with infarcts in multiple arterial territories was reported in 43% of cases.^2^ The majority of strokes are ischemic with only approximately 10% being hemorrhagic. The most common stroke mechanism was found to be cryptogenic in 45%, followed by cardioembolic in 22%, large vessel atherosclerotic in 11% and small artery stroke in only 3%.^2^ COVID associated with stroke was associated with a much higher mortality and increased disability, although this may partly reflect the predominance of large artery strokes and the fact that stroke in COVID-19 patients was often associated with severe COVID-19 infection.^2^

## How might COVID-19 increase stroke risk and does this have implications for therapy?

Mechanisms underlying stroke in patients with COVID-19 are likely to be multifactorial. In some patients they could be related to conventional stroke mechanisms, with COVID-19 acting as a trigger, and this is consistent with conventional risk factors such as hypertension, diabetes mellitus, and coronary artery disease being associated with increased stroke risk in individuals with COVID-19 infection.^2^ However, in others stroke may be directly caused by COVID-19 infection through specific pathophysiological mechanisms. Activation of the coagulation pathway, with elevated D-dimer and fibrinogen, which have been reported in patients with COVID-19 and stroke, has been suggested as one mediating pathway.^2^ This coagulopathy, related to the infection-induced systemic inflammatory response, may contribute to the risk of both stroke by casing both arterial thrombosis, and also venous thrombosis with paradoxical embolism. This may explain why large vessel occlusion is seen in some younger individuals who have no vascular risk factors. Anticardiolipin antibodies have also been reported in some patients with COVID-19, and may relate to increased thrombosis risk, although more data are required.^4^ A characteristic feature of severe COVID-19 infection is the “cytokine storm”. This describes uncontrolled activation of the immune system caused by the viral infection with subsequent excessive cytokine release, which may itself result in thrombosis, as well as plaque rupture and endothelial injury. Direct myocardial injury has also been described including myocardial dysfunction related to the cytokine storm, and viral myocarditis, and these may lead to cardiac arrhythmias and intracardiac thrombus, possibly exacerbated by the hypercoagulable state, and therefore cardioembolic stroke.^5^

Direct viral invasion has also been suggested as a disease mechanism. COVID-19 uses the angiotensin-converting enzyme 2 receptor to enter cells. This receptor is expressed in vascular endothelium as well as heart, lungs, and kidneys and direct invasion of endothelial cells causing an “endotheliitis” has been proposed as one mechanism contributing to the thrombotic complications of COVID-19.^2^ Although acute lacunar stroke appears less common in COVID-19 infection radiological changes of cerebral small vessel disease including cerebral microbleeds have been reported.^6^ It has been suggested these could result from both endothelial injury precipitated by the mechanisms above, and also hypoxemia.

The increased risk of thrombosis, including venous thrombosis,^7^ has led to the recommendation that all hospitalized patients with COVID-19 should receive prophylactic heparin.^8^ It has been hypothesized that full dose anticoagulation may also reduce thrombosis in small arteries in multiple organs including the lungs, and therefore could reduce disease severity and the need for ventilatory support. Early trials suggested no benefit of full anticoagulation in COVID-19 patients on ventilators, but more recent data suggest a possible benefit in patients with less severe COVID in reducing progression of respiratory disease.^9^ Whether such approaches will reduce the frequency of thrombotic events such as stroke remains to be determined. However, such approaches are likely to have a limited effect on reducing stroke incidence associated with COVID because the majority of patients with stroke and COVID present with the stroke itself rather than developing stroke while in hospital; 84% in a recent systematic review.^2^

## The impact of the pandemic on stroke services

Initial monitoring by the WSO revealed widespread effects of the pandemic on delivery of stroke services.^1^ Most countries saw significant service reorganization, and WSO members reported reallocation of neurology and stroke beds including intensive care facilities to COVID-19 patients, necessitating a move of stroke units to less optimal accommodation, as well as redeployment of stroke physicians, nurses, and other stroke healthcare related workers to the care of COVID-19 patients. Since these early observations, numerous studies have documented these effects. A meta-analysis of nine studies in 59,233 subjects reported a drop to 64% in the number of stroke alerts, a drop to 69% in reperfusion therapies, and a drop in of mechanical thrombectomies to 78%.^10^ These effects appear to be global. A recent large collaborative study between the Society of Vascular and Interventional Neurology, the Middle East and North Africa Stroke and Interventional Neurotherapies Organisation, the Japanese Interventional Neurology Society, and academic partners from six continents and 40 countries reported a global decline in the volume of overall stroke hospitalizations, mechanical thrombectomy procedures, and intracranial hemorrhage admissions.^11^ These reductions were observed regardless of COVID-19 hospitalization burden and pre-stroke and mechanical thrombectomy volumes. Declines in stroke hospitalizations and MT volumes were if anything greater in mid and high volume versus low volume stroke centers, perhaps related to the fact that larger centers are more likely to become the preferred destination for COVID-19 referrals due to capacity issues.

Reassuringly COVID-19 patients do seem to be receiving reperfusion therapies despite the challenges delivering them during the pandemic. In one meta-analysis, the ratio of the number of mechanical thrombectomies to stroke patients was reported to be higher than usual with an odds ratio of 1.23, perhaps reflecting the increased incidence of large artery occlusion associated with COVID-19 infection. A more recent meta-analysis including 18 cohorts studies involving 67 845 patients reported that COVID-19 infection was not associated with any alteration in the chance of receiving intravenous thrombolysis (IVT; OR 1.42, 95% CI 0.65–3.10) or thrombectomy (OR 0.78, 95% CI 0.35–1.74).^12^

The reasons for this reduction in activity have been debated. A major factor is likely to have been reduced presentation of patients with stroke, particularly with milder symptoms, due to concerns about the risk of catching COVID-19 infection in hospital.^13^ Physical distancing measures may also have prevented patients suffering from a stroke being witnessed at that time. Another alternative could be reduced hospital access for COVID-19 patients, although international studies have suggested this was unlikely to be the case. Whether the incidence of stroke itself has altered remains uncertain. It has been suggested that the environmental consequences of a lockdown, with improved patient behavior and medication compliance might reduce vascular events, while a reduction in exposure to other common viruses, or even reduced levels of pollution, might result in reduced triggering of vascular events including stroke risk. On the other hand, COVID-19 appears to be causally related to stroke at least in some cases.^2^ A recent population based study reported that despite this reduction in stroke presentations there was actually an increase in total stroke deaths, but that most of these occurred in the community at home and particularly in nursing homes, while in hospital stroke deaths fell.^14^ We await longer term population based studies to fully understand the true effect of the pandemic on stroke incidence.

## Adapting stroke services

In response to the pressure on stroke services, numerous National and International guidelines have been developed both on the web and as publications.^15–18^ Algorithms have been developed for optimal acute stroke care to ensure rapid diagnosis of COVID infection in stroke patients, protection of patients and healthcare staff, and optimal practices to maintain delivery of reperfusion services both thrombolysis and thrombectomy. The importance of telemedicine has been widely recognized. Tele-stroke networks were already implemented worldwide for delivery of thrombolysis, but these practices have been extended to help provide acute stroke care during the pandemic.^19^ Adaptions have been made to deliver stroke care in low resource setting, including those caused by the pandemic. For example, an emerging concern in low-resource environments is whether the monitoring intensity of a subpopulation of acute ischemic stroke patients undergoing treatment with IVT can safely be reduced. A low-intensity monitoring protocol for patients treated with thrombolysis in whom the NIH Stroke Scale was less than 10 at the time of stroke presentation, was successfully piloted.^20^ and is now being evaluated in the International OPTIMIST trial in 12000 participants.^21^ A global campaign (#StrokeDontStayAtHome) was created by the WSO in collaboration with the Angels Initiative to raise awareness of the need for people to call an ambulance and seek immediate medical attention if they suspect stroke. (https://www.world-stroke.org/world-stroke-day-campaign/world-stroke-campaign/strokedont-stay-at-home)

The pandemic has also challenged provision of outpatient stroke care and rehabilitation. Many transient ischemic attack (TIA) services have been switched to virtual with the use of either telephone or video telemedicine technology, often with surprisingly good results. Telemedicine approaches have also been suggested for some rehabilitation services; this does limit the range of rehabilitation that can be provided, although a recent randomized controlled trial showed encouraging results for delivery of home-based motor training telerehabilitation program on motor function in patients with stroke.^22^ Many patients have received less rehabilitation both due to earlier discharge both to free up hospital beds and avoid risk of in hospital infection, and reduced availability of community rehabilitation services. There have been concerns that this could have long-term consequences on stroke outcome. Guidelines have been produced for the longer-term restoration and recovery of stroke services.^23^

## Does stroke increase the risk of severe COVID-19 infection?

An initial concern to our patients was the possibility that previous stroke could increase the severity of COVID-19 infection. An early meta-analysis suggested there was a 2.5 fold increase in the risk of severe COVID-19 in those with a history of cerebrovascular disease.^24^ A similar odds ratio has been reported in more recent larger studies.^25^ However, much of this appears to be related to comorbidities. For example, a more recent study showed markedly higher risks of hospitalization (32.4% vs. 11.7%) and death (13.4% vs. 3.6%) in COVID-19 patients with prior stroke.^25^ However, after matching for demographics and comorbidities these differences were markedly reduced with the difference in rate of hospitalization falling to 32.3% versus 26.0%, and the death rate not being significantly elevated (13.4% vs. 12.9%). This suggests that much of the increased risk of severe COVID-19 infection associated with a past history of stroke is explained by comorbidities including vascular risk factors such as hypertension, diabetes, and obesity which are known to increase the risk of severe COVID-19, and disability resulting from the stroke. The consequences of COVID-19 infection have been reported to be particularly severe in individuals with dementia, whether this results from a neurodegenerative or vascular cause.^26^

A number of stroke patients ask whether it is safe to take the COVID vaccine. There is no evidence of any specific risk in stroke patients, and public health bodies are advising that stroke patients should receive it.

## Inequalities

Stroke is already an unequal disease. Sociodemographic factors are an important risk factor for stroke and there are widespread geographical disparities in stroke incidence and outcome. While stroke incidence appears to be reducing in many higher income countries, in many low and middle income countries it is increasing.^27^ These striking inequalities have been exacerbated by COVID-19. Socioeconomic status, education, and ethnicity are risk factors for mortality secondary to COVID.^28^ Preventable conditions including cardiovascular disease and type 2 diabetes are themselves major risk factors for dying from COVID-19^29^ and these disproportionally effect people living in disadvantaged areas and from ethnic minority backgrounds. A survey of stroke survivors in Brazil during the pandemic found 59% were in financial difficulties, 48% had difficulties getting food, and 42% had appointments with doctors cancelled (unpublished data, Brazilian Stroke Network Survey).

The pandemic has emphasized the deep inequalities and differences in life expectancy that exist between different groups within individual countries, and between countries. There is a danger that the economic consequences of the pandemic will further exacerbate global inequalities. Only National governmental and International initiatives can address these issues.

## Impact of the pandemic on stroke research

**Figure 1.**
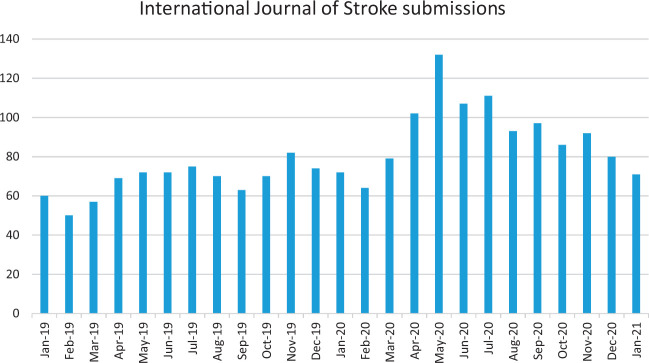
Submissions per month to the *International Journal of Stroke*, showing a marked increase during the initial lockdown.

The pandemic has had a major impact on stroke research. Initially most stroke journals saw an unexpected increase in submission. *The International Journal of Stroke*, the WSO flagship journal, received 58% more submissions from April to June 2020 compared with the previous 4 months (Figure 1). This presumably reflected “catch-up” writing during the lockdown. However, the long-term consequences have been more worrying. Charitable research income has been badly hit resulting in many schemes funding stroke research being temporarily halted. As an example, the Association of Medical Research Charities in the UK’s research sector, which invested £1.9 billion in medical research in 2019, had a 38% loss in fundraising income.^30^ Seventy percent of all UK clinical trials and studies were paused in April 2020 and 54% remained paused in June.^30^ The impact on charitable research funding is likely to remain for some time, and countries will be dependent on governmental funding being maintained, or even increased, to make up this shortfall. The long-term economic consequences of the pandemic may also reduce governmental funding.

The long period of lockdown and restricted working during the pandemic has had a major impact on young researchers, which has been particularly severe for female researchers with childcare responsibilities.^31^ There has been much discussion as to how funding and other bodies can mitigate these consequences. There has been a proliferation of research articles on COVID-19, and a corresponding exponential increase in articles examining its association with stroke. Many of these have been of poor quality and the expedited review processes implemented have sometimes been less rigorous. There have been a large number of papers which have been subsequently retracted and articles have highlighted the `carnage of substandard research' related to COVID-19.^32^ However, conversely the pandemic has led to a sharing of expertise and impressive collaboration and streamlining of processes for clinical trials. For example, the RECOVERY series of trials that may be beneficial in patients with COVID-19 have recruited over 35,000 participants.^33^ In Brazil, a research network for COVID-19 was created (COALITION) including 92 hospitals, resulting in rapid evaluation of treatments including the perspective of a middle income country highly affected by the disease.^34^ These collaborative ventures have led to rapid assessment of the benefits, or lack of benefits of a number of agents. Similarly progress in developing, evaluating and regulatory approval for COVID vaccines has been impressive.

## Telemedicine in clinical stroke care and research—A possible benefit of the pandemic

Perhaps the biggest benefit for stroke care may be the recognition of the benefits of telemedicine. While telemedicine was already widely used for acute stroke assessment, particularly in geographically dispersed areas, the pandemic has expanded this use. It has highlighted its potential in diverse aspects of stroke care, many of which may be beneficial after the pandemic. It also revealed how unprepared many health services were for its delivery both in terms of equipment and technical familiarity of healthcare professionals.^19^ During the pandemic successful application has been described in TIA clinics,^35^ physical, occupational, and speech therapy rehabilitation,^36^ and nursing communication with families.^37^ The benefits of video technology, allowing limited examination and an awareness of the patient’s expression, over telephone consultation have been demonstrated.^38^ It was often suggested that many stroke patients, being elderly, would not manage well with the technology but video technology has been shown to be well adopted to by outpatients with stroke and cognitive impairment.^38^ Even in non-pandemic times this technology offers major advantages to patients who can have their consultations while at work or in their own home, and avoid often lengthy travel times. The potential of telemedicine in performing assessments in clinical trials has also been appreciated.^39^ Perhaps these advances are something we can take forward into the future.
